# Reliability of a Novel Video-Based Method for Assessing Age-Related Changes in Upper Limb Kinematics

**DOI:** 10.3389/fnagi.2018.00281

**Published:** 2018-09-24

**Authors:** Daniel A. Pupo, John W. Kakareka, Jonathan Krynitsky, Lorenzo Leggio, Tom Pohida, Stephanie Studenski, Brandon K. Harvey

**Affiliations:** ^1^Translational Gerontology Branch, Intramural Research Program, National Institute on Aging, National Institutes of Health, Baltimore, MD, United States; ^2^Signal Processing and Instrumentation Section, Office of Intramural Research, Center for Information Technology (CIT), National Institutes of Health, Bethesda, MD, United States; ^3^Section on Clinical Psychoneuroendocrinology and Neuropsychopharmacology, National Institute on Alcohol Abuse and Alcoholism (NIAAA) and National Institute on Drug Abuse, National Institutes of Health, Bethesda, MD, United States; ^4^Center for Alcohol and Addiction Studies, Brown University, Providence, RI, United States; ^5^Optogenetics and Transgenic Technology Core, Intramural Research Program, National Institute on Drug Abuse, National Institutes of Health, Baltimore, MD, United States

**Keywords:** upper extremities, video-based monitoring, reliability, aging, kinematics, fiducial markers

## Abstract

Monitoring age-related changes in motor function can be used to identify deviations that represent underlying diseases for which early diagnosis is often paramount for efficacious, interventional therapies. Currently, the availability of cost-effective and reliable diagnostic tools capable of routine monitoring is limited. Adequate diagnostic systems are needed to identify, monitor and distinguish early subclinical symptoms of neurological diseases from normal aging-associated changes. Herein, we describe the development, initial validation and reliability of the Hand-Arm Movement Monitoring System (HAMMS), a video-based data acquisition system built using a programmable, versatile platform for acquiring temporal and spatial metrics of hand and arm movements. A healthy aging population of 111 adults were used to evaluate the HAMMS via a repetitive motion test of changing target size. The test required participants to move a fiducial on their hand between two targets presented on a video monitor. The test-retest reliability based on Intraclass Correlation Coefficient (ICCs) for the system ranged from 0.56 to 0.87 and the Linear Correlation Coefficients (LCCs) ranged from 0.58 to 0.87. Average speed, average acceleration, speed error and center offset all demonstrated a positive correlation with age. Using an intertarget path of hand motion, we observed an age-dependent increase in the average number of points outside the most direct motion path, indicating a reduction in hand-arm movement control with age. The reliability, flexibility and programmability of the HAMMS makes this low cost, video-based platform an effective tool for evaluating longitudinal changes in hand-arm related movements and a potential diagnostic device for neurological diseases where hand-arm movements are affected.

## Introduction

Aging is the primary risk factor for developing neurodegenerative diseases. Early diagnosis of neurodegenerative diseases is paramount to effective therapies and there is a substantial effort to develop non-invasive medical devices to identify early changes that may reflect a disease or disorder. Movement disorders often occur in underlying neurologic or muscular degenerative diseases, such as Parkinson’s disease (PD), amyotrophic lateral sclerosis (ALS) and muscular dystrophy (MS; Ketcham and Stelmach, 2004; Jiang and Dickson, 2018). The average age of people has led to an increasing population of people 65 years and older and this is projected to keep increasing. The increasing population of older people will lead to a higher prevalence of neurological disease and an increased need for treatment (Tysnes and Storstein, 2017). Early detection of these diseases could be one of the keys to addressing this growing issue. The devastating impact on people with movement disorders and their families has led to the development of tests and devices to detect movement disorders earlier in life. Several methods have been developed to monitor movement disorders that are associated with upper extremity dysfunction such as spiral test (Haubenberger et al., 2011; Jeonghee et al., 2016; Legrand et al., 2017), wearable accelerometers (Golan et al., 2004; Wang et al., 2017), video-based system (Ugbolue et al., 2013; Filippeschi et al., 2017). These devices can effectively measure upper body movement kinematics, yet accelerometers produce data with high signal-to-noise ratios, creating difficulty in data extraction, which leads to issues with the reliability (Wong et al., 2007; Hyde et al., 2008). The Hand-Arm Movement Monitoring System (HAMMS) described herein is an alternative video-based system that is designed to be simple, low cost and reliable in terms of use, data collection and data processing.

High medical expenses necessitate the development of cost-effective diagnostic methodologies, especially as the human lifespan increases and requires more medical attention. The economic burden for PD, alone is projected to double by the year 2040 so there will be a need for affordable diagnostic methods (Kowal et al., 2013). Age-related neurological diseases often progress to a point of limited therapeutic efficacy once obvious changes in movement have been diagnosed. Imaging-based testing—such as MRI, CT and PET scanning—can be used to identify a pathology, but these techniques are often employed in response to symptoms that present at later stages of a disease. Routine *in vivo* neuroimaging can be used to detect physiological trends that indicate early onset of disease, but this route is cost prohibitive. Other non-imaging techniques identify molecular biomarkers in tissue or fluids and have has been utilized in PD detection (Rosenthal et al., 2016; Arshad et al., 2017). Although genetic predispositions and altered biomarkers can indicate the vulnerability to and presence of early stage disease, these screenings are not routinely performed. Instead, a patient’s family history and overt symptoms are primary triggers for testing biomarkers or imaging. Overall, these approaches are costly, present a multitude of limitations/challenges and are typically only useful in identifying diseases at late stages (Ciurleo et al., 2014). For these reasons, precise low-cost methods for early disease detection need to be developed to aid an aging population by implementing therapies designed to slow or halt disease progression. Therefore, objective, low-cost diagnostic systems, such as the HAMMS, are necessary to justify utilizing more sophisticated and high-cost techniques when patients lack symptoms that are reflective of late-stage diseases.

Control of movement is an interaction between both sensory and motor systems. As people age, motor function declines as impaired central processing reduces the ability to optimally coordinate extremities (Seidler et al., 2010). Most studies have examined gait to better understand age-related changes in coordination (Gimmon et al., 2015), with a few studies looking at coordination in upper limb movement. Studies of upper body coordination suggest that a loss of cognitive capacity with aging leads to a loss in the ability to process sensorimotor information (Rey-Robert et al., 2012). When testing repetitive motion tasks that involve both a speed and accuracy component, young people have been shown to be faster than older people. For example, in one study the older participants were more focused on accuracy than speed, possibly due to a lack of ability to process the task as rapidly as the young participant, in turn compensating by focusing more on accuracy (Ketcham et al., 2002; Ketcham and Stelmach, 2004). Most studies have focused on the ability to reach higher speeds and maintain that the participants reach the highest speeds possible while being as accurate as possible. There have been a few studies that look at how controlled movement changes with age with many studies focused on the gait and lower extremity control rather than upper extremity (Gimmon et al., 2015).

In this article, we describe the HAMMS, a novel technique that uses a minimally invasive method and affordable computerware to monitor goal-directed repetitive hand movements over a widely aged group of individuals. The HAMMS is shown to be a reliable clinic-friendly diagnostic system that is low cost, reliable and could be used to execute quick routine examination for movement disorders.

## Materials and Methods

### Participants

This was a cross sectional study evaluating the reliability of the HAMMS in human subjects using participants from the Baltimore Longitudinal Study of Aging (BLSA; Shock et al., 1984) total, 191 subjects performed the HAMMS tests, although 80 were removed from the study because of an identified motor issue, missing data from the Ideomotor Apraxia test, injury to the dominant hand, or had left-handed dominance, as well as technical issues with data acquisition. The final number of participants was 111 right-handed participants (52 women, 59 men, mean age 72 ± 12, age range 43–94). BLSA participants have an intense daily schedule that consists of performing other tests during their visit, which required that the second trial for reliability assessment test be conducted after a medical interview lasting 1 h after the initial trial (i.e., 1-h break between trials).

The study protocol was approved by the NIEHS Institutional Review Board. All participants provided written consent following the guidelines and practices of the Baltimore Longitudinal Study on Aging and the National Institutes of Health, set by the Declaration of Helsinki.

### System Overview

**Figure 1:**
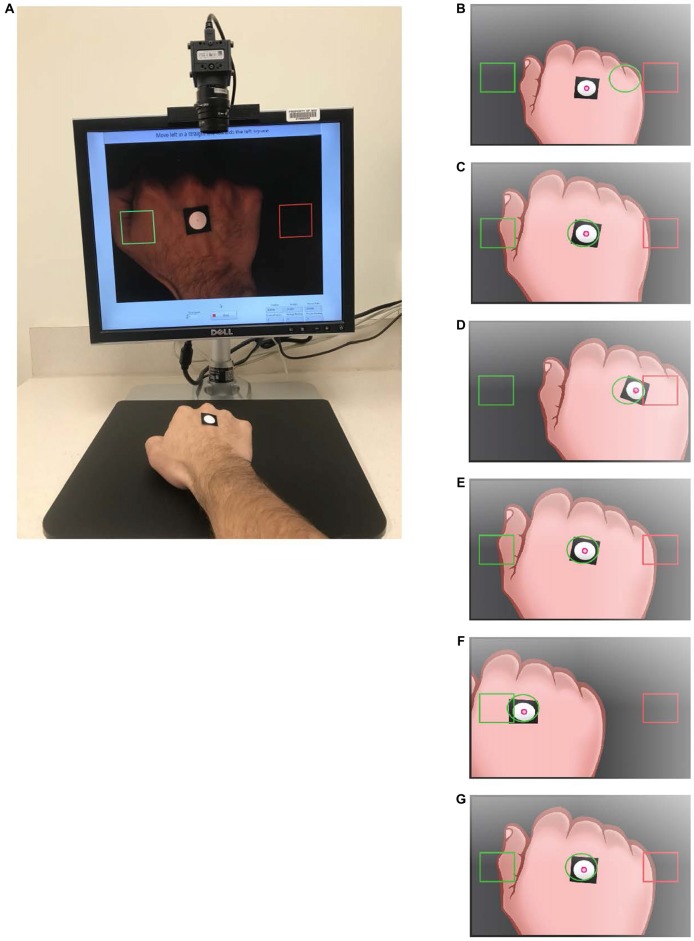
The Hand-Arm Movement Monitoring System (HAMMS). **(A)** Overview of the HAMMS, a video-based hand tracking system that uses the centroid of circle calculated in real-time to monitor hand movement. An adhesive black square with white circle is placed on the dorsal surface of participant’s hand and the software recognizes the circle and creates a centroid (red dot) which is tracked and provided as feedback with 30 frames every second. During training, a guide circle (green) appears on screen **(B)**. The participant aligns the white circle with the green guide circle **(C)** and then follows the guide between the two square targets to establish motion and expected speed **(D–G)**.

Designed to be a routine and low-cost system, the HAMMS monitors simple hand motions using video analysis to provide real-time feedback to the participant. Labview software (National Instruments, Austin, TX, USA) was used to generate the user interface, real-time camera feedback and data acquisition/processing (see Supplementary Methods for detailed information). The participant sat in front of a computer monitor connected to a downward-facing camera, with an 18 × 18-mm adhesive fiducial marker (a stark black and white 12-mm contrast circle) placed on the back of their hand, of which the participant was instructed to guide through superimposed target squares (Figure 1A). The HAMMS components were selected to minimize cost while allowing flexibility in trial design. The system consisted of a USB 3.0-camera (IDS UI1220SE-C-HQ, Obersulm, Germany), lens (Kowa LM8JC, Torrance, CA, USA), custom mounting hardware, and a consumer-grade Windows 7 desktop and monitor (Dell Optiplex 990 and Dell 1708 fpt, Round Rock, TX, USA). The camera mount consisted of a 3D-printed monitor bracket, an 11-cm stainless-steel beam, and a 3D-printed angle bracket, all to ensure that the camera maintained a fixed location in relation to the monitor. The monitor’s height was adjusted to 380-mm from the camera to the tabletop, which provided a desired camera field-of-view of 183-mm. Encompassing the field-of-view, a rectangular piece of black plastic was placed on the tabletop to prevent reflections from interfering with video processing.

### Software Information

The LabVIEW (National Instruments, Austin, TX, USA) program was comprised of a calibration sequence followed by five concurrent runtime loops, and included: frame acquisition, fiducial marker recognition, target square processing, segmentation, data storage, region of interest processing, and progression of the trial sequence. At the beginning of each trial, a calibration step was performed where the participant maintained the fiducial marker on their hand in a displayed square, which enabled the acquisition of a single calibration frame. The calibration frame was processed to find the minimum and maximum thresholds that can be used for detecting the white circle on the marker, with a median value stored as the threshold for all subsequent real-time processing. Once the calibration step concludes, the user begins the training.

During runtime, the camera acquires frames at 30 frames per second (fps) which were saved to a buffer by the frame acquisition loop. The fiducial marker recognition loop reads frames from the buffer, segments frames using the calibration threshold and uses circle detection to discriminate the fiducial marker from artifacts. The program extracts the circle radius and centroid of the fiducial marker from the segmentation results and forwards the information to a data buffer along with the raw frame. The program uses the centroid information to determine whether the fiducial marker is within any of the displayed target squares and updates status variables for each target square. This loop also overlays a red dot at the detected centroid position on the live video displayed to the participant as feedback. A separate target square processing loop uses the centroid information to determine whether the fiducial marker is within any of the displayed target squares, and updates status variables for each target square. The data storage loop loads the segmentation results and raw images from the data buffer and saves the experiment data (centroid location and status information) to a text file. Raw images are saved to an AVI file for visual verification and debugging. The subtask sequence loop monitors the target square status variables to determine completion of portions of the subtasks (i.e., if the user reached an endpoint) and progresses the subtask sequence accordingly. Finally, the region of interest loop monitors the status variables for each displayed target square and updates each region’s color to help provide visual feedback to the user (green until the participant reaches the target then the target becomes red). Additionally, this loop monitors the current step in the subtask sequence and changes the displayed target squares accordingly.

### Movement Task

**Figure 2:**
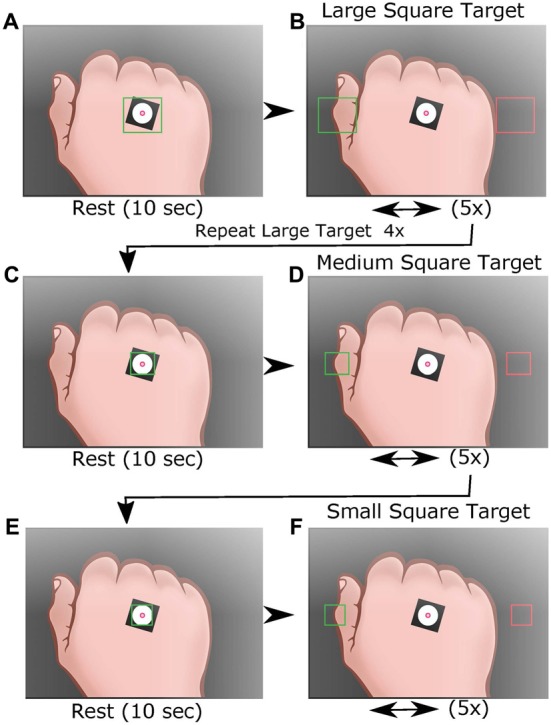
Display for the HAMMS test. Following training, participants are asked to move the white circle to the target square, then rest arm for 10 s **(A)**. Participants are asked to move the white circle to the center of the left large target square (green) to initiate the first task of five movements between the two large target squares **(B)**. After five movements the participants return to rest **(A)**, then repeats the task three times (4× total for the large target squares). After a 10 s rest **(C)**, participants move the white circle to the center of the left medium target square (green) to initiate five movements between the two medium target squares **(D)**. A final task is conducted using small square targets **(E,F)**. The location of the centroid is recorded throughout all six tasks. Each participant performed two trials of the six tasks with an hour of separation between trials.

**Figure 3:**
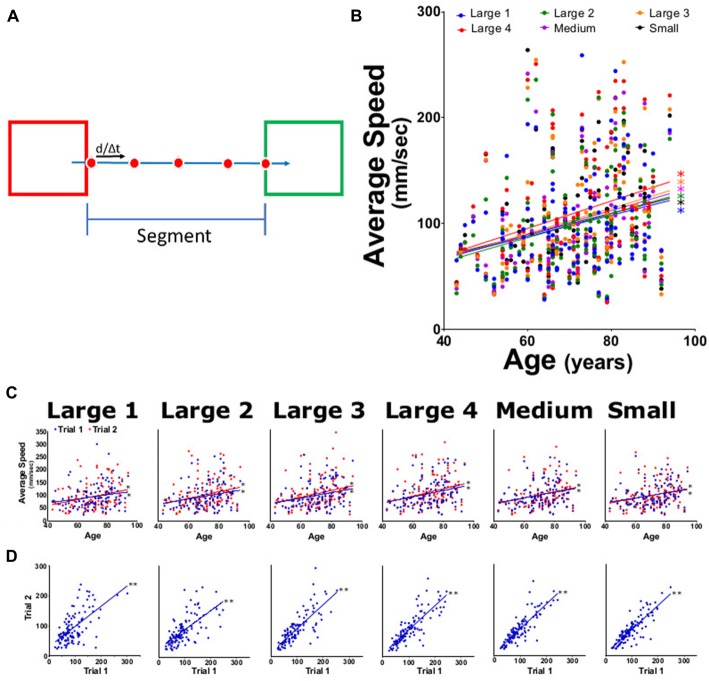
Average speed for each task. **(A)** Diagram of speed calculation using distance between centroid locations across two camera frames (red dots) at 0.033 frames/s. Speed was averaged across the span of a segment for each subtask (five segments per target subtask). **(B)** Averaged segment speed for each task (large 1–4, medium and small targets) for both trials as function of participant age. **(C)** Average speed from trial 1 (blue) and trial 2 (red) tasks per participant age. **(D)** Linear regression analysis of trial 1 vs. trial 2 speed for each task. *p < 0.05, **p < 0.001.

Participants were introduced to the system via verbal instructions (see Supplementary Methods for the script) and were asked to “maintain a constant steady pace,” to practice the motion between target squares using a moving guide circle to establish an approximate speed of 25.7 mm/s for the trial (Figures 1B–G). During the motion, the hand lightly glided over the tabletop to help the participant maintain a constant height. The training continued until the administrator determined that the participant could sufficiently maintain the desired speed, after which the guide circle disappeared from the screen. Following the practice session, a resting screen consisting of a single large 100 × 100-pixel-centered square indicated that the participant could rest (i.e., place their hand in a comfortable position within the square) for 10 s before the trial commenced (Figure 2A). After the resting screen, the display showed two horizontally aligned large target squares (100 × 100 pixels) at fixed locations on either side of the display, centered vertically (Figure 2B). The left and right squares were green and red, respectively, at the beginning of the trial. The participant moved their hand to the green square, which turned red when the centroid entered the square. The opposing square then turned green and the participant moved to that square. One movement between squares was defined as a travel segment, which was the path taken after leaving one target square and arriving at the next. An endpoint segment was defined as the path taken from entering the target square and reversing direction (Figure 3A). Repeating the process five times was a subtask (Figures 2A,B), which was then followed by a resting screen for 10 s. The subtask was repeated four times with large targets, once with a medium square (62 × 62 pixels), and once more with a small square (50 × 50 pixels; Figures 2C–F). Respectively, the subtasks were referred to as Large 1, Large 2, Large 3, Large 4, Medium 1 and Small 1, corresponding target square size and repetition number. The completion of all three configurations of target squares (six subtasks total) was a trial. In total, each participant completed two trials with a standard 60-min BLSA medical questionnaire separating the two trials. An example video of a single trial is provided (Supplementary Video S1).

### Data Analysis

**Figure 4:**
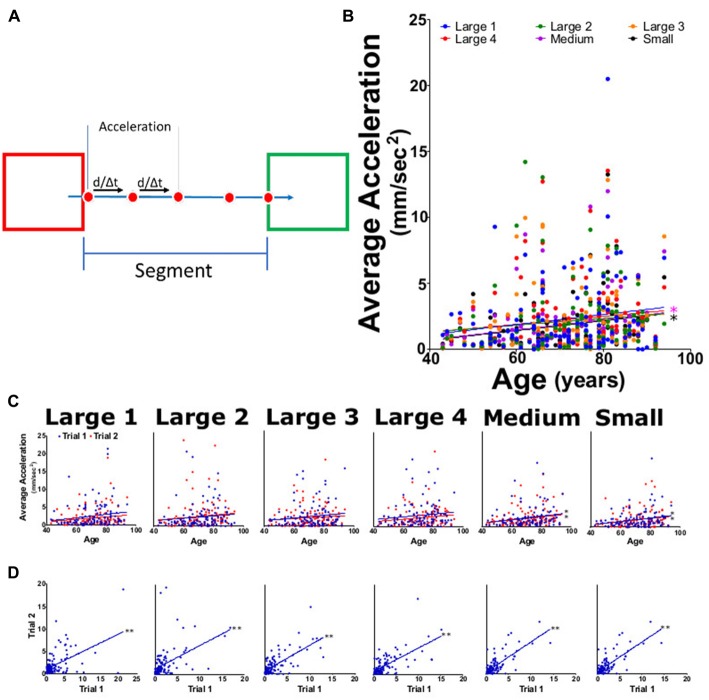
Average acceleration for each task. **(A)** Diagram outlining acceleration calculations where the speed between four frames was used to calculate acceleration and averaged across the entire segment. **(B)** Average task acceleration for large 1–4, medium, and small targets for both trials as function of participant age. **(C)** Average acceleration from trial 1 (blue) and trial 2 (red) tasks per participant age. **(D)** Linear regression analysis of trial 1 vs. trial 2 acceleration for each task. *p < 0.05, **p < 0.001.

**Figure 5:**
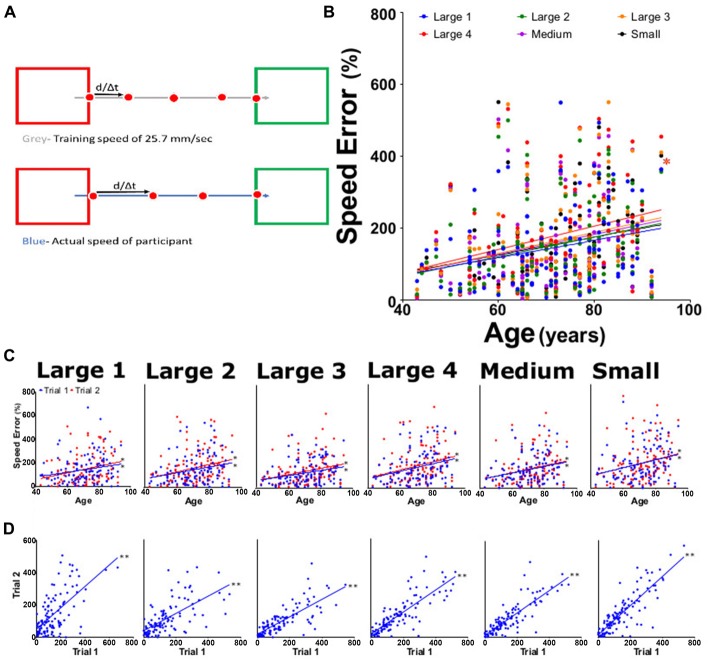
Percent speed error measurements. **(A)** Speed error was calculated by subtracting the training speed (gray; a constant at 25.7 mm/s) from the actual measured speed (blue), then dividing by training speed. **(B)** Average percent speed error for large 1–4, medium, and small targets for both trials as function of participant age. **(C)** Average percent speed error from trial 1 (blue) and trial 2 (red) tasks per participant age. **(D)** Linear regression analysis of trial 1 vs. trial 2 of offset for each task. *p < 0.05, **p < 0.001.

**Figure 6:**
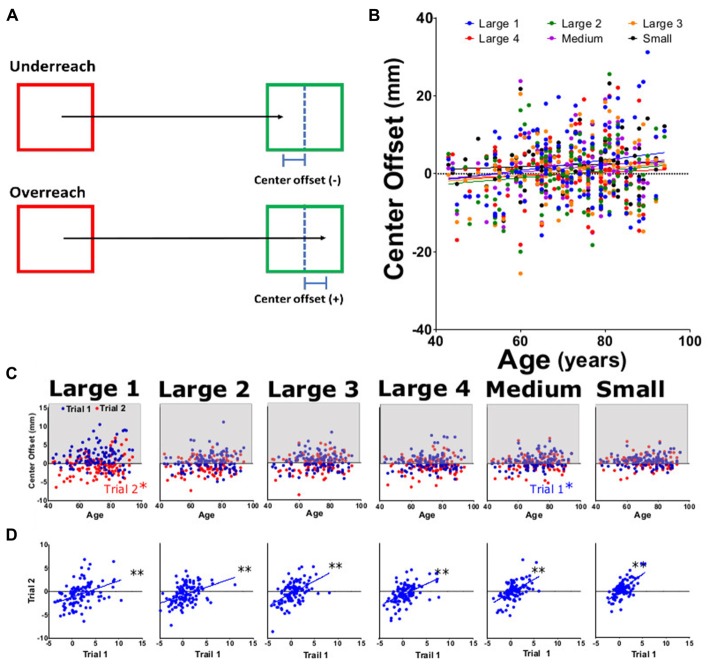
Center offset for approximated target. Center offset was measured as the distance by which the participant over- or undershot the center of the target square. **(A)** Diagram of center offset. **(B)** Average center offset for large 1–4, medium, and small targets for both trials as function of participant age. **(C)** Average center offset from trial 1 (blue) and trial 2 (red) tasks per participant age. **(D)** Linear regression analysis of trial 1 vs. trial 2 of offset for each task. *p < 0.05, **p < 0.001.

**Figure 7:**
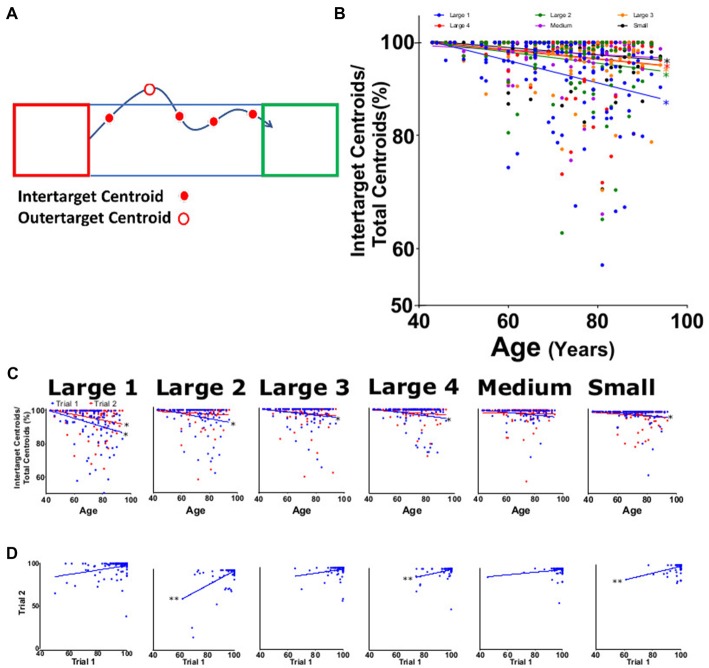
Intertarget path accuracy (IPA). **(A)** Diagram showing measurement of movements within specified target path (blue rectangle between target squares). As depicted, four of the five total centroids captured were within the rectangle (solid red dots) resulting in 80% IPA. **(B)** Average IPA for large 1–4, medium, and small targets for both trials as function of participant age. **(C)** Average IPA from trial 1 (blue) and trial 2 (red) tasks per participant age. **(D)** Linear regression analysis of trial 1 vs. trial 2 of offset for each task. *p < 0.05, **p < 0.001.

Upon completion of each trial, the HAMMS saved a text file containing the centroid location and target status information for each frame collected during the trial. The study used five types of metrics: (1) speed, calculated as the instantaneous speed for each travel segment (Figure 3A), where endpoint segments were excluded; (2) acceleration, calculated as the instantaneous acceleration for each travel segment (Figure 4A), again excluding endpoint segments; (3) speed error, calculated as the amount of change from the attempted controlled speed of 25.7 mm/s (Figure 5A); (4) center offset, using the endpoint segments to measure the horizontal distance (left-to-right) from the center of a target square and the farthest point reached by the participant (Figure 6A). A negative number (underreach) indicated that the participant did not reach the center of the target before reversing direction, while a positive number (overreach) indicated that the participant went past the center of the target before reversing direction; and (5) intertarget path accuracy (IPA), measured how the participants stayed within a given region of interest, defined as a rectangular region on the screen with a height matching the target square in each large, medium and small subtask (100, 62, 50 pixels, respectively) and a width which fully encompasses both target squares (Figure 7A). Travel outside this region indicated failure to maintain sufficient control. The number of frames that the centroid was outside this region was divided by the total number of frames for a given subtask to normalize for different travel speeds.

### Statistical Analysis

For the statistical analysis, all travel segments for a subtask were averaged together. Correlations between parameters with age were determined using linear regressive models, which were performed using the averaged results between both trials for all square sizes, as well as for each individual trial. Statistical significance was determined as *p* value < 0.05. The Intraclass Correlation Coefficient (ICC) was used to assess the reliability of the system using each of the parameters measured, which were further confirmed by calculating Linear Correlation Coefficients (LCCs) and Confidence Intervals. Significant correlations were determined as *p* value < 0.001. ICC was calculated using a two-way fixed effects model (ICC (2, k)) calculated from outputs run in a Two-Way ANOVA without replication (GraphPad Prism v7.00; Walter et al., 1998; Hoenig et al., 2017; Muyor, 2017).

## Results

### Speed

The HAMMS is a simple video-based device that was designed to track participants moving between two squares on the monitor in order to detect possible changes in kinematics with age. For each participant, all recorded segments from a subtask were averaged together and a linear regression model was calculated using the averaged results for both trials. Linear regression models for average speed and average acceleration increased as a function of age (positive slope values) for all subtasks of the test. When compared against age, all subtasks for speed were shown to be significantly increased (Figure 3B). The slope values for speed ranged from 0.999 to 1.143 (Figure 3B). Both trials 1 and 2 for speed showed statistical significance, with age-dependent upward trends for the different subtasks (Figure 3C), slopes ranging from 0.7961 to 1.389. A significant, positive correlation between trial 1 and trial 2 for speed was observed (Figure 3D), indicating that participants demonstrated less control in the second trial. The reliability measurements for speed were: ICC values of 0.85 (0.79 to 0.90) and LCC values of 0.87 (0.82–0.90; Table 1).

**Table 1:** Intraclass correlation coefficient (ICCs) and linear correlation coefficients (LCCs), both with confidence intervals of 95%.

	Speed	Acceleration	Center offset	Intertarget path accuracy	Speed error
ICC (95% CI)	0.86 (0.80–0.90)	0.85 (0.79–0.89)	0.61 (0.20–0.80)	0.56 (0.41–0.67)	0.84 (0.77–0.89)
LCC (95% CI)	0.87 (0.82–0.90)	0.85 (0.79–0.90)	0.72 (0.62–0.80)	0.58 (0.44–0.69)	0.85 (0.79–0.90)

### Acceleration

For the statistical analysis, all segments for a task were averaged together. A linear regression was calculate using the averaged results for the acceleration of both trials (Figure 4B). Acceleration was seen to follow a slight upward trend showing a slight positive increase in age with each target size, slopes for each task were shown to have values that ranged from 0.013 to 0.038 (Table 2). A significant upward trend with age was seen for only medium and small tasks (Figure 4B; *p* values of 0.024 and 0.013, respectively). Individual tasks for trials 1 and 2 showed the same positive trend with age and had slopes ranging from 0.027 to 0.047 for trial 1 and from 0.019 to 0.036 for trial 2 (Figure 4D); only tasks medium and small showed significant trends in trials 1 and 2 (Figure 4C; *p* < 0.05) When comparing trial 1 against trial 2 (Figure 4D; *p* < 0.001) each task showed a highly significant positive increase in the acceleration. The reliability measurement for this parameter had an ICC of 0.85 (0.79–0.89) and an LCC of 0.85 (0.79–0.90; Table 1).

**Table 2:** Slope and *p* values for the trial 1 and trial 2 averaged results of each size.

	Speed	Acceleration	Center offset	Intertarget path accuracy	Speed error
Large 1 Slope (*p* Value)	0.999 (0.0052)*	0.038 (0.0816)	0.138 (0.0533)	−0.212 (0.0008)*	0.024 (0.0051)*
Large 2 Slope (*p* Value)	1.137 (0.0019)*	0.026 (0.1992)	0.093 (0.1197)	−0.105 (0.0393)*	0.027 (0.0025)*
Large 3 Slope (*p* Value)	1.194 (0.0023)*	0.025 (0.1915)	0.091 (0.1359)	−0.082 (0.0311)*	0.029 (0.0027)*
Large 4 Slope (*p* Value)	1.295 (0.0018)*	0.030 (0.1226)	0.052 (0.3722)	−0.085 (0.0438)*	0.032 (0.0015)*
Medium Slope (*p* Value)	1.143 (0.0021)*	0.038 (0.0240)*	0.083 (0.1072)	−0.044 (0.2240)	0.028 (0.0026)*
Small Slope (*p* Value)	1.013 (0.0050)*	0.013 (0.0130)*	0.031 (0.4895)	−0.070 (0.0321)*	0.023 (0.0048)*

*P< 0.05.

### Speed Error

All participants moved with an average speed that was faster than 25.7 mm/s, with speed errors ranging from 5% to 500%. An upward trend in the speed error was seen in all subtasks for each trial, with slopes ranging significantly from 0.023 to 0.032 (Figure 5B). This trend continued when the data was evaluated for each subtask from both trial 1 and trial 2 (slopes 0.020 and 0.034, respectively; Figure 5C). The individual subtasks between trial 1 and trial 2 also showed a significant positive increase with age (Figure 5D). Reliability for speed error was 0.84 (0.77–0.89) for ICC and 0.85 (0.79–0.90) for LCC (Table 1).

### Center Offset

We examined the path of motion using two approaches. The first was target center offset; an index of overreach/underreach from the estimated target center. No significant differences were seen for center offset across subtasks (Figures 6B,C) with slope values ranging from 0.031 to 0.138 (Table 2). When comparing center offset trial 1 to trial 2, each was significantly correlated (Figure 6D), indicating that patients were consistently overshooting/undershooting the estimated center of the square. Examining individual trials by center offset data (Figure 6C), trial 1 medium and trial 2 large had significant positive trends with age, while upward trends for other subtasks were non-significant. Reliability analysis for this motion path parameters showed ICC and LCC values for center offset as 0.61 (0.41–0.67) and 0.72 (0.62–0.80), respectively.

### Intertarget Path Analysis of Movement

IPA was measured as a decrease in the percentage of the number of frames in which the centroid is inside of the most direct regional path (Figure 7A). The IPA for the combined trials significantly decreased with age for all target sizes except medium (*p* = 0.22), with slopes ranging from −0.044 to −0.212 (Figure 7B; Table 2). When separated by trial and subtask, all IPA trends were negative, but not all were significant. Slopes were seen to be significant and negative in trial 1 with values ranging from −0.078 to −0.256 and not significant for trial 2 values ranging from −0.010 to −0.168 (Figure 7C, *P* < 0.001). When examining consistency between trials, IPA showed significant correlations for large 2, large 4 and small (Figure 7D). Reliability for this parameter was the lowest of all the HAMMS parameters with an ICC of 0.56 (0.41–0.67) and LCC values of 0.58 (0.44–0.69).

## Discussion

### Performance and Reliability of the HAMMS

Using a video-based monitoring system, we demonstrated data consistency across 111 participants (Table 1). Using the wide range of ages for participants, all physically and mentally healthy, the reliability measures were interpreted to be an accurate representation of the system’s ability to observe spatial and temporal aspects of upper extremity movement. Participants from the BLSA are very healthy from the general aging public and as such, in our opinion, represent an appropriate group to test the reliability and validity of the study.

System reliability is commonly tested over days/weeks, but this is not possible with BLSA participants, as they return at varying times based on age. Instead, participants were evaluated with an hour break between trials to test reliability, which has been previously observed as a valid methodology (Unver et al., 2015a,b). The number of participants collected is considered adequate to give an accurate representation of the device performance and the obtained ICC values were at least 0.4 (Walter et al., 1998). Based on the minimal criteria for a reliable clinical device, the HAMMS produced reliable ICC and LCC values for all measured metrics, matching the criteria for a reliable clinical device (Table 1). Stronger ICC values were obtained for the temporal metrics of speed, acceleration and speed error (0.86, 0.85 and 0.84, respectively) compared to spatial metrics of IPA and center offset (0.56 and 0.61, respectively), although these values still fell within an acceptable range. Our results show the tests are reliable and identify age-related changes in spatial and temporal parameters.

### Age-Related Changes in Kinematic Metrics

Motor control typically diminishes with age (Yan et al., 1998; Verrel et al., 2012). In this study, a healthy sample of BLSA participants, ranging in age from 43 to 94, were asked to maintain a constant speed in a repetitive hand motion task. Participants in the BLSA are considered healthy participants based on study criteria and any participants with potential confounds (e.g., tremors, injuries) were excluded from the HAMMS study to keep the analyzed sample a group of healthy individuals differing in age.

Examining 111 healthy participants with the HAMMS, speed error was observed to increase with age, suggesting an age-related loss in speed control. Although younger participants still had an average of ~100% error in the task (average speed was twice the desired speed of 25.7 mm/s), older participants ranged upwards of 600% error within the same subtask. There was an observed significant change in speed error as a function of age for all subtasks, and this change in speed was consistent between trials. This increase in speed may be due to age-associated factors, such as decreased short-term memory deficits regarding the desired motion speed, but further testing is needed.

Despite training for the test at a desired speed, age-dependent changes were also observed in speed-related metrics and in spatial-related metrics (center offset and IPA), although to a lesser extent. Consistent with these observations, older participants showed more consistent overreach compared to younger participants. LCC curves revealed that participants improved their ability to approximate and reverse direction closer to the center of the target squares across trials (Figure 6D). This improved accuracy is likely due to experience gained from trial 1 to trial 2, but further research is required. A similar trend was observed for IPA and the trial 1 vs. trial 2 plots suggested that as, participants progressed through the target sizes, a higher number of points were obtained within the most direct regional path. However, there remained an age-related trend of decreased accuracy with older participants. Previous work using the Fitts task has shown both young and old participants improve with training (Boyle et al., 2015) and we saw evidence of improvement between the two trails. Our results are also consistent with previous studies that found older participants have more variability in movement and difficulty maintaining a constant trajectory due in part to impaired spatial reasoning (Ketcham and Stelmach, 2004). Overall, our data support that the HAMMS was able to identify an age-related effect on intertarget centroid movement.

The HAMM system requires that participants employ many different neural pathways for proper execution. Anatomical areas involved in completing the test include areas dealing with motor control (e.g., motor cortex and basal ganglia), visual control (e.g., visual cortex) and areas of the frontal lobe dealing with executive functions. The completion of the HAMMS task involves planning that may shift the relative contributions of basal ganglia and frontal cortex (Heuninckx et al., 2008). For example, if there is more planning than execution, one might expect participants to have higher speed error with less center offset which is what we observed in older patients. More studies are needed to elucidate the relative contribution of the various pathways and anatomical regions responsible for each measured parameter (i.e., speed, acceleration, center offset and IPA). An advantage of HAMMS is that it serves a more general detector of pathway dysfunction while allowing for flexibility in design to ultimately target specific components of the nervous system.

### Design Limitations

Since the HAMMS was designed to be low cost and clinic friendly, there are some limitations in system performance. PC hardware can limit data transfer from the camera, as well as the computing power needed for real-time video analysis. To minimize system costs, the camera can only achieve a maximum of 30 frames per second. Moving at the desired 25.7 mm/s speed, with a center-to-center target distance of 138 mm, the system acquires ~166 frames for each travel segment, but the video frame rate of the current the HAMMS has been used in past studies to evaluate changes in hand motion (Castellote et al., 2004; Smeragliuolo et al., 2016). The limitations in frame rate and computing capabilities of the HAMMS restricts participant speed, which helps ensure acquisition of sufficient data points for analysis. Future upgrades to the HAMMS should include higher frame rates, which would allow for faster movement speed acquisition and analysis.

Another design limitation is video processing of the fiducial marker. Theoretically, fiducial marker analysis could be developed to monitor vertical hand motion by measuring the size of the fiducial, as experienced when the hand moves up towards the camera, causing the fiducial to appear larger. However, due to the large working distance of the lens and limitations in the fiducial recognition algorithm, the fiducial size could not be measured with sufficient accuracy to achieve appropriate resolution of the vertical hand position. Therefore, participants were instructed to move just above the tabletop to maintain a constant vertical position, which reduced error in other calculations due to unknown motions in Z. Using this configuration, hand movements could be reliably measured in the X-Y plane.

### Future Research Directions

This study established the HAMMS test as a reliable means of video-based data acquisition and analysis of hand-arm motion. We evaluated the system using BLSA participants to examine a healthy, diverse patient population of various ages. A major benefit of integrating the HAMMS within the BLSA is the repeated testing on the same participants over extended time intervals. Future testing of the HAMMS using the same participants would confirm its utility in early disease detection, disease progression and treatment efficacy, but is beyond the scope of the current study. A short-term objective is to evaluate the HAMMS using patient populations with muscular or neurological diseases that involve upper extremity dysfunction (i.e., PD, Friedreich’s ataxia and stroke), specifically both before and during treatment. However, by identifying age-related changes in hand-arm kinematics in a healthy population, we can begin to look for deviations as an indicator of other non-neurological diseases as well. For example, by having established a set of parameters that change as part of normal, healthy aging, the HAMMS could be used as standard test during a routine checkup to look for deviations that may reflect a pathological state. It will also be interesting to see whether baseline HAMMS may predict treatment response and/or the HAMMS-related parameters change over time in response to the medical treatments. Finally, the ultimate long-term goal of this research is to assess the potential for the HAMMS to detect early-onset muscular or neurological diseases at subclinical stages.

Further software development for directed tasks could improve the utility of the HAMMS. For example, pseudo-randomized ordering of target size may augment task difficulty. Hardware changes, such as increasing camera speed and resolution, would allow more detailed analyses for both task-oriented and natural hand motions. Finally, the effects of task learning on motor control could be evaluated by changing target shape and location in repeating and non-repeating sequences.

Overall, the HAMMS system performed reliably when tested on healthy aging population, identifying an age-dependent effect in speed error and intertarget centroid accuracy. By establishing age-related changes to upper limb kinematics in healthy individuals, deviations that represent underlying pathological states in aged adults may ultimately be identified using HAMMS.

## Author Contributions

DP collected and analyzed data and drafted the manuscript. JKakareka, JKrynitsky and TP designed the system, developed algorithms to process the raw data and edited the manuscript. LL provided the initial study sample, guided the system design and edited the manuscript. SS provided study sample and financial backing for the study, designed the study and edited the manuscript. BH initiated the project, designed and guided the study design, analyzed data and edited the manuscript.

## Conflict of Interest Statement

The authors declare that the research was conducted in the absence of any commercial or financial relationships that could be construed as a potential conflict of interest.
