# Supplementary material for: Reliability of a Novel Video-Based Method for Assessing Age-Related Changes in Upper Limb Kinematics
Source: Front Aging Neurosci. 2018 Sep 24;10:281. doi: 10.3389/fnagi.2018.00281 (PMC6166023; doi:10.3389/fnagi.2018.00281)
Supplement: Supplementary file 1 [file Presentation_1.PDF]

Supplemental Methods:

Script: "The following task will consist of six trials; four easy, one medium and one hard. Please rest your elbow on the arm rest and extend your arm so the target sticker is in the center of the screen. For each trial, you will be moving your hand from the left box to the right box and back several times while trying to change direction in the center of the box. Always move toward the box that is highlighted in green. When you reach that box, a sound will chime and it will turn red while the opposite box will turn green indicating it is the new destination. The goal of the task is to aim for the center of the target as closely as possible, while also maintaining a constant steady pace. Try to keep the motion fluid and direct. While moving, try to keep your hand level with the marker facing up and try to keep the circle on the screen. Also, please try to keep your elbow on the arm rest using that location as a pivot point. Between each trial, there will be a 10 second rest period before the next trial. You can now practice on the screen several times."
